# Bacterial Diversity of Water and Soil Samples from Dripping Springs in the Organ Mountains (New Mexico), Determined by Using 16S rRNA Gene Amplicon Sequencing

**DOI:** 10.1128/MRA.01079-19

**Published:** 2019-11-21

**Authors:** John A. Kyndt

**Affiliations:** a College of Science and Technology, Bellevue University, Bellevue, Nebraska, USA; Georgia Institute of Technology

## Abstract

Dripping Springs is located on the western side of New Mexico’s Organ Mountains. This is an initial microbial analysis of the spring water and surrounding microbial mat. Both soil-water and water samples showed a high level of microbial biodiversity consistent with the secluded and pristine nature of the area.

## ANNOUNCEMENT

The Dripping Springs natural area contains a wealth of habitats with great biological diversity, including four wildflower species endemic to the area, the endangered Organ Mountains evening primrose, and other rare plants (https://www.blm.gov/visit/dripping-springs-natural-area) (1); however, no studies have been reported on the microbial diversity in the area or of the spring waters. The spring water runs into a small mountain rivulet found in a narrow, secluded canyon. The water source and its surrounding granite wall have often been described as “magical,” and the area was an ideal setting for a tuberculosis sanatorium in the late 19th to early 20th century (see https://www.blm.gov/visit/dripping-springs-natural-area and https://www.desertusa.com/desert-new-mexico/ruins-dripping-springs.html). Largely secluded from anthropogenic influences, the spring is expected to be a sanctuary of microbially diverse populations.

To obtain a snapshot of the bacterial composition, we isolated samples from the spring runoff in July 2019. One sample was taken from the water dripping across the rock surface (DPSI), while a second soil-water sample included some of the microbial mat that covers the rock surface (DPSII) (32°19′23.34″N, 106°34′24.39″W). Samples were collected in sterile 15-ml collection tubes, stored in a cooler with ice packs for ∼2 h, and transferred to the lab, where they were stored at 4°C. The total DNA was extracted using the PureLink microbiome DNA purification kit (Invitrogen). Utilizing a Qubit fluorometer and NanoDrop spectrophotometer, we determined the quality and quantity of DNA, showing 260/280 ratios of 1.75 (DPSI) and 1.90 (DPSII), respectively. A 16S rRNA amplicon sequencing library was prepared for each sample, following the 16S rRNA metagenomic sequencing library preparation protocol (Illumina). Amplicon primers targeting the V3 and V4 regions were synthesized by Sigma (2). The samples were sequenced using a 1.8 pM library with an Illumina MiniSeq system. Paired-end (2 × 150-bp) sequencing generated 1,479,192 reads for DPSI and 1,592,048 reads for DPSII. The primer sequences were removed, and reads with low-quality scores (average score, <20) were filtered out using the FASTX-Toolkit within BaseSpace (version 2.2.0; Illumina). The 16S Metagenomics app within BaseSpace (version 1.0.1) was used to perform taxonomic classification, which uses an Illumina-curated version of the Greengenes taxonomic database and the RDP Naive Bayes taxonomic classification algorithm (3). Default parameters were used for all software applications, unless otherwise noted.

**FIG 1:**
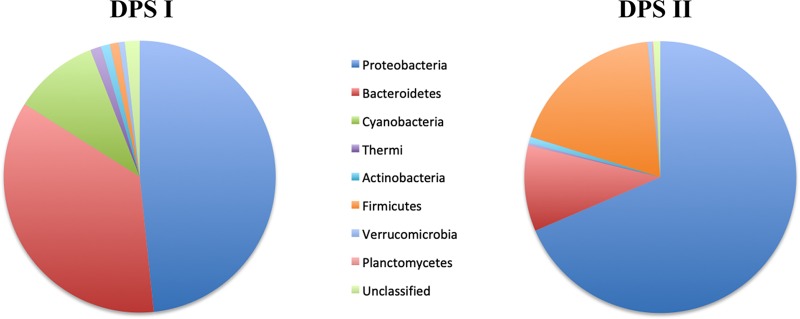
Pie charts of bacterial diversity based on 16S rRNA gene amplicon analysis of samples from Dripping Springs at the phylum level. DPSI is a surface water sample, while DPSII is a soil-water mixed sample.

Figure 1 illustrates the most abundant operational taxonomic units (OTUs) in the taxonomic classifications at the phylum level. In DPSI, the most abundant were *Proteobacteria* (47.81%), *Bacteroidetes* (35.2%), and *Cyanobacteria* (10.0%). In contrast, in DPSII, *Proteobacteria* (68.4%), *Firmicutes* (18.7%), and *Bacteroidetes* (10.2%) were found most abundantly. For DPSI and DPSII, 81.3% and 93.0% of the reads, respectively, were classified to the genus level. Even though the two samples were isolated from the same runoff area, there were substantial differences between the soil-water (DPSII) and water (DPSI) samples. In general, cyanobacteria and other photosynthetic bacteria had a higher representation in DPSI, while the families *Pseudomonadaceae* and *Clostridiaceae* were exclusively found in the DPSII soil-water sample.

According to a Shannon species diversity analysis (run within the 16S Metagenomics app) (4, 5), each of the samples contained over 1,000 potential species, with significant differences between the soil-water and water isolates. In addition, both samples contained 40 to 60% unidentified reads at the species level, which opens up possibilities for further studies on microbiological diversity and ecological impacts.

### Data availability.

The 16S rRNA gene amplicon data sets have been deposited at DDBJ/ENA/GenBank under the accession number PRJNA561623 and can be accessed with the SRA accession numbers SRR10017618 (DPSI) and SRR10021907 (DPSII).
